# Many lessons still to learn about autosomal dominant polycystic kidney disease

**DOI:** 10.1007/s44162-023-00017-8

**Published:** 2023-09-01

**Authors:** Sarah Orr, John A. Sayer

**Affiliations:** 1 Translational and Clinical Research Institute, Faculty of Medical Sciences, Newcastle University, Central Parkway, Newcastle Upon Tyne, NE1 3BZ UK; 2 Renal Services, Newcastle Upon Tyne Hospitals NHS Foundation Trust, Newcastle Upon Tyne, NE7 7DN UK; 3 NIHR Newcastle Biomedical Research Centre, Newcastle Upon Tyne, NE4 5PL UK

**Keywords:** ADPKD, Penetrance, Prevalence, Whole Exome Sequencing, Rare Disease

## Abstract

We are still learning the genetic basis for many rare diseases. Here we provide a commentary on the analysis of the genetic landscape of patients with Autosomal Dominant Polycystic Kidney Disease (ADPKD), one of the most common genetic kidney diseases. Approaches including both phenotype first and genotype first allows some interesting and informative observations within this disease population. *PKD1* and *PKD2* are the most frequent genetic causes of ADPKD accounting for 78% and 15% respectively, whilst around 7–8% of cases have an alternative genetic diagnosis. These rarer forms include *IFT140*, *GANAB*, *PKHD1*, *HNF1B*, *ALG8*, and *ALG9*. Some previously reported likely pathogenic *PKD1* and *PKD2* alleles may have a reduced penetrance, or indeed may have been misclassified in terms of their pathogenicity. This recent data concerning all forms of ADPKD points to the importance of performing genetics tests in all families with a clinical diagnosis of ADPKD as well as those with more atypical cystic kidney appearances. Following allele identification, performing segregation analysis wherever possible remains vital so that we continue to learn about these important genetic causes of kidney failure.

In the UK and Europe, a rare disease is defined as a disease that affects less than 1 in 2000 individuals [23]. In the USA, the Food and Drug Administration (FDA) defines a rare disease as a disease which affects less than 200,000 Americans [1] (which works out to be around 1 in every 1600 individuals).

Autosomal dominant polycystic kidney disease (ADPKD) is the most common genetic cause of kidney failure and accounts for between 2.5 and 10% of all patients globally needing dialysis or a kidney transplant [15]. In the US, it is the fourth most common cause of end stage renal disease (ESRD) [6]. The molecular genetics of ADPKD are well described with *PKD1* variants contributing to around 78% of cases and *PKD2* alleles contributing to 15% of cases and the remainder unsolved [16]. However, more recent genetic studies have identified a set of additional genes that may cause ADPKD accounting for most of this missing 7% [19].

Whether or not ADPKD is a rare disease remains a point of contention among the scientific community, as there is much debate over the incidence of ADPKD due to differences in screening and how the disease is diagnosed. For symptomatic cases, ADPKD meets the European definition of a rare disease [17]. However, it is thought that a large proportion of patients with ADPKD remain undiagnosed throughout life but may be identified post-mortem, via autopsy [10]. For example, in Japan 1998, patients in hospital estimated a peak prevalence of 261/million for ADPKD (~ 0.5 cases in 2000), enabling it to be classified as a rare disease for symptomatic cases [10]. Early clinical studies of ADPKD focussed on cases identified post-mortem with autopsy cases investigated in both Denmark and Minnesota. These studies estimated the incidence of ADPKD to be around 1 in 400– 1 in 1000 live births, which becomes too common for classification as a rare disease [3]. An autopsy study in Hong Kong Chinese individuals published in 1993 found ADPKD in 1 in 339 autopsies in Hong Kong compared to 1 in 503 in Western countries [4], both of which classify ADPKD as too frequent for a rare disease. However, these historical studies involving autopsies can now be seen as potentially inaccurate due to the inability to distinguish between ADPKD and other cystic diseases and acquired cysts in patients with chronic kidney disease (CKD). This may have led to an overestimation in the prevalence of ADPKD.

Epidemiological studies have also been employed to estimate the incidence of ADPKD. An epidemiological study in France estimated the prevalence to be around 1 in 1111 [3, 20]. By tracking all patients accessing nephrology services in Alentejo in Portugal, the incidence of ADPKD was estimated to be around 1 in 3019 [7]. Studies outside of Europe include an investigation in the Seychelles. All doctors in the Seychelles were asked to refer all confirmed and suspected ADPKD patients for systemic examination, including of haplotypes and family history [27]. The overall incidence was around 57/100,000 (~ 1.1 in 2000) [27]. However, the majority of cases were in Caucasian individuals, with a prevalence in this population of 1 in 542 [3, 27], with very few cases in individuals of Black descent. This could be due to a founder effect amongst the Caucasian population in the Seychelles, skewing the results. More recent epidemiological studies in Europe estimate that the ADPKD prevalence is below the threshold for a rare disease in Europe, with an estimated prevalence of between 2.41/10,000 and 4.6/10,000 depending on whether population-based, screening or registry-based methods were used [25]. This incidence is reflected in epidemiological studies in the USA. Willey et al. estimated that around 140,000 patients are currently diagnosed with ADPKD each year in the USA [26], below the less than 200,000 threshold set by the FDA for classification as a rare disease. There has also been reported differences in incidence in different ethnicities in the USA, which is contrary to that previously reported in the Seychelles [3, 27], as it has been reported that the prevalence of ADPKD is highest in Black patients in the USA, with an incidence of 73 per 100,000 [2]. The overall incidence was around 4.3/10,000 [2], reflecting the European prevalence. However, the large disparity in prevalence between ethnic groups in the US highlights the importance of assessing a large racially and ethnically diverse group when determining the incidence of a rare disease. Detailed studies in the USA also indicate that the prevalence of ADPKD varies between different regions of the USA based on availability of diagnostic tools in each area [24]. Access to care should therefore also be considered when estimating the global prevalence of ADPKD. One study in Olmstead County, Minnesota in the USA estimated a higher incidence of ADPKD point prevalence of 6.8/10,000 [22], however genetic testing will be required to confirm the genetic cause of these potential ADPKD cases. This highlights the importance of genetic testing for heterogeneous rare diseases such as ADPKD.

More recent studies have focused on genetic testing and genetic screening of family members. A clinical study of germline mutation screening for ADPKD-genes *PKD1* and *PKD2* in a population of German patients recruited by both nephrologists and non-nephrologists all of whom presented with ADPKD-type features found an overall prevalence of 32.7/100,000 (~ 0.6 in 2000) individuals, meeting the criteria for a rare disease [18]. The authors also concluded that non-genetic based studies led to an overall overestimation in ADPKD cases. A similar study was carried out using genetic screening in Modena, Italy to confirm diagnosis of ADPKD following MRI and ultrasound scans [21]. Taken in conjunction with analysis of published literature about European prevalence of ADPKD, the authors concluded ADPKD had a 3.63/10,000 point prevalence and a 4.76/10,000 predicted prevalence [21], meaning that in European populations it is a rare disease [21]. In the age of whole exome and whole genome sequencing, it is likely we will be able to define the prevalence of ADPKD more accurately across different population groups.

The recent paper by Chang et al. utilises whole exome sequencing technology, using an unselected health system-based cohort (of mainly European descent) and found in a cohort of 174,172 patients with a median age of 60 years, 303 patients had ADPKD based on ICD-9/10 diagnoses (~ 3.48 in 2000) [5].

Via analysis of the whole exome sequencing of their ADPKD population, Chang et al. describe a combined prevalence of likely pathogenic and pathogenic *PKD1* and *PKD2* alleles of 8.64 per 1000 [5]. Looking at pathogenic alleles alone, this decreased to 1.93 per 1000 [5]. By examining known mutations alongside the ICD9/10 codes, the incidence became 1.74 per 1000 [5].

The Chang paper builds upon work previously carried out by Lanktree et al. in 2018 [13] by combining whole exome sequencing data analysis with clinical records [5]. By uniquely differentiating between and utilising two separate approaches, genotype first and phenotype first, more information could be uncovered [5]. The unique findings include that some previously classified likely pathogenic variants in *PKD1* and *PKD2* are actually not pathogenic at all due to a lack of disease phenotype or a high wild type population allele frequency [5]. This potential for misclassification of *PKD1* and *PKD2* alleles was also reported by Lanktree in 2018 [13], as 17.3/10,000 individuals in this multi-racial cohort were found to carry missense variants which were classified as ‘likely pathogenic’ suggesting a misclassification of these variants, highlighting the complexity involved in classifying missense variants. Hence, caution should be used when classifying missense variants as potentially causative, as clinical phenotype should be assessed alongside genotype.

By utilising pedigree segregation data, many newly discovered variants uncovered via exome sequencing were found to be likely pathogenic, further uncovering the complexity and heterogeneity of this disorder [5]. The paper highlights the need for the integration of various approaches and of phenotypic and genotypic data when investigating diseases such as ADPKD with many different genetic causes.

Before discussing the penetrance of various ADPKD mutations, it is important to define the difference between penetrance and expressivity. Penetrance refers to the presence of a clinical phenotype in an individual, while expressivity describes differences in phenotype between individuals carrying the same genotype [28]. Different ADPKD mutations exhibit different levels of penetrance, i.e., genetic causes of atypical ADPKD causes generally have a lower and more variable penetrance than *PKD1* or *PKD2* mutations [9]. This is an example of reduced penetrance rather than low expressivity, as the level of clinical phenotype in these cases tends to remain consistent between individuals with the same molecular genotype.

Wider literature has previously defined the penetrance of truncating *PKD1* and *PKD2* mutations to be 100% (Fig. 1) [3, 9]. This is interpreted to mean that any individual who inherits a pathogenic mutation in these genes will develop a kidney cyst by age 30, which is detectable by ultrasound [11]. Factors including severity of their disease, the age of onset of the cyst and the extra-renal manifestations they present with do vary between patients [11]. Non-truncating *PKD1* and *PKD2* mutations have a much more variable penetrance (Fig. 1) [9]. Non-truncating *PKD1* mutations uncovered by exome sequencing alone and not-confirmed in clinical populations tend to be incompletely penetrant, with a mild if any phenotype. However, those uncovered in clinical populations, and confirmed by co-segregation with phenotype in pedigrees, such as the HALT PKD trial are fully penetrant and can be equally as severe and significant as truncating variants [14]. Occasionally, the ages of the clinical manifestations for non-truncating *PKD1* variants also match those with truncating *PKD1* mutations [14]. There is a low frequency of non-truncating *PKD2* mutations within populations of ADPKD patients. Chang et al. report a 100% penetrance of ADPKD in patients with confirmed *PKD1* loss of function mutations, consistent with previous literature [5]. For *PKD2* the authors concluded that all patients with *PKD2* large deletions or loss of function mutations had an ADPKD diagnosis following imaging analysis, but this was not reflected in the ICD9/10 terms [5], indicating the need for genetic testing and image review rather than just relying on ICD9/10 terms for an accurate diagnosis of ADPKD. The authors also reported that just 31% of individuals with likely pathogenic *PKD1* missense mutations had clinically diagnosed ADPKD [5], and several likely pathogenic variants had multiple unaffected carriers [5] suggesting a greatly reduced penetrance, or once again misclassification of *PKD1* missense variants. At the individual level, a patient with a *PKD2* likely pathogenic mutation was confirmed to have ADPKD once their chart was reviewed but did not according to ICD9/10 terms alone.

**Fig. 1:**
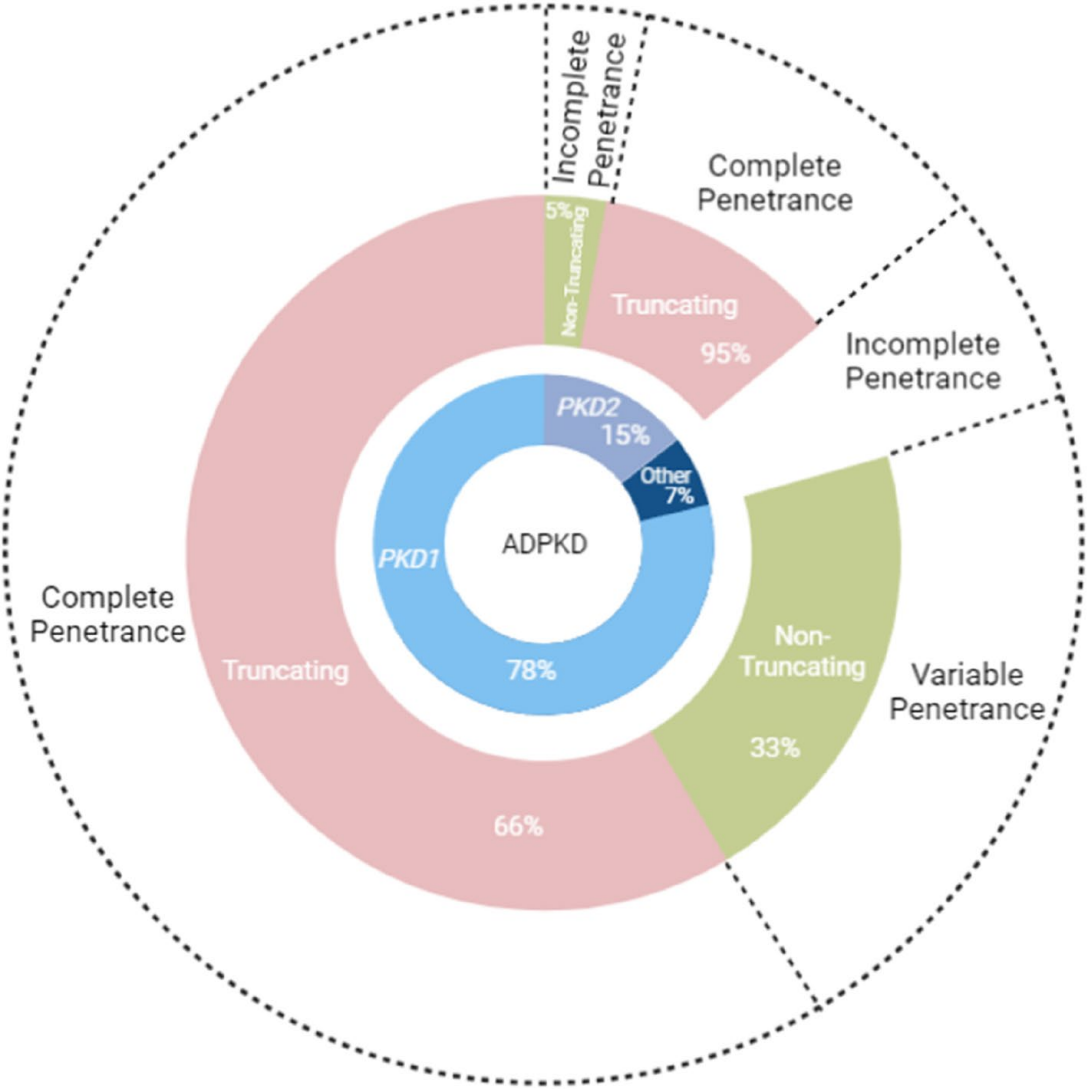
Sunburst plot summarising the reported genetic landscape of ADPKD and the reported disease penetrance of different subtypes of *PKD1* and*PKD2* variants and of 'Other’ variants. ‘Other’ variants refers to atypical causes of ADPKD such as *ALG8*, *ALG9*, and *IFT140* which are discussed in the manuscript, highlighting the genetic heterogeneity of this disorder. The penetrance of non-truncating *PKD1* variants has been given as ‘Variable’ due to the differences in penetrance of these variants depending on whether they were uncovered via exome sequencing alone, or within clinical populations and confirmed by co-segregation. This summary integrates information from many of the studies discussed in the manuscript and was created using BioRender

Atypical ADPKD causing genes tend to have a much more variable penetrance (Fig. 1) [9], for example *ALG9* is known to have a reduced penetrance compared to other atypical PKD genes [9]. Chang et al. found 8.1% of patients with ADPKD in a phenotype first analysis had a rare variant in a gene associated with atypical ADPKD [5]. These genes were *IFT140*, *GANAB, PKDH1, HNF1B, ALG8*, and *ALG9* [5]. Rare variants were only identified in 11 of 23 cases of atypical ADPKD following patient stratification [5]. These patients were much more difficult to genetically ‘solve’ due to the variable penetrance of atypical PKD genes. Furthermore, information about penetrance of alleles in these genes is not fully reliable in these patients, as the authors were unable to review images or chart information for all of these patients. The variable penetrance and milder phenotype reported in the atypical cases is reflected in previous literature regarding atypical ADPKD.

Lanktree et al. reported in 2021 that many genes relevant to both atypical ADPKD and Autosomal Dominant Polycystic Liver Disease (ADPLD) are involved in the endoplasmic reticulum biosynthetic pathway and should be considered when investigating the genetic cause of kidney or liver cysts in a patient [12]. Variants within genes such as *DNAJB11* have been shown to result in atypical ADPKD phenotypes [12]. Rare variants in genes such as *PKRSCH, SEC61B* and *SEC63* have been attributed to ADPLD, but should also be considered when making a molecular genetic diagnosis in patients with kidney cysts due to the interlinking nature of the pathogenesis mechanism of kidney and liver cysts, resulting from perturbed post-translational polycystin-1 modification, reducing functional polycystin-1 levels [12]. These findings further highlight the genetic heterogeneity and complexity of ADPKD, and the list of minor genes causing atypical ADPKD will likely increase following more detailed molecular genetic investigations into the causes of ADPKD. Evidence to date suggests that heterozygous loss of function variants in *IFT140* account for the third most common form of ADPKD [19] but we are still learning about these atypical causes.

The Chang et al. paper is the most recent of a series of papers documenting the genetic landscape of ADPKD patients. This paper highlights the importance of whole exome sequencing in combination with clinical data to accurately diagnose and estimate the prevalence of rare diseases such as ADPKD, especially in more diverse populations to re-classify missense variants that are labelled as ‘likely pathogenic’ but are not producing a disease phenotype. Furthermore, looking at just ICD9/10 codes alone cannot confirm a ADPKD diagnosis, and radiological review and genetic tests will be needed to fully confirm a precise diagnosis. Thus, genetic tests should be integrated with detailed phenotypic review to confirm an ADPKD diagnosis. The debate surrounding ADPKD as a rare disease thus continues! It is likely that wider genetic testing would detect more benign cases of ADPKD, increasing its incidence and leading it to be redefined as a common disease. However, as we begin to reclassify ADPKD into its genetic subtypes such as the work being performed by ClinGen [8], we can begin to understand ADPKD subtype by genotype, such as ADPKD-*PKD1*, ADPKD-*PKD2*, ADPKD-*IFT140* etc. and define personalised approaches to this set of rare diseases.

## Data Availability

Data sharing not applicable to this article as no datasets were generated or analysed during the current study.
